# Robust Determination of Fatigue Crack Propagation Thresholds from Crack Growth Data

**DOI:** 10.3390/ma15144737

**Published:** 2022-07-06

**Authors:** Josef Arthur Schönherr, Larissa Duarte, Mauro Madia, Uwe Zerbst, Max Benedikt Geilen, Marcus Klein, Matthias Oechsner

**Affiliations:** 1 Center for Structural Materials (MPA-IfW), Technical University of Darmstadt, 64283 Darmstadt, Germany; max.geilen@tu-darmstadt.de (M.B.G.); marcus.klein@tu-darmstadt.de (M.K.); matthias.oechsner@tu-darmstadt.de (M.O.); 2 Bundesanstalt für Materialforschung und -Prüfung (BAM), Division 9.4, 12205 Berlin, Germany; larissa.duarte@bam.de (L.D.); mauro.madia@bam.de (M.M.); uwe.zerbst@bam.de (U.Z.)

**Keywords:** fatigue crack propagation threshold, ISO 12108, ASTM E647, data evaluation methods, experimental determination

## Abstract

The robust determination of the threshold against fatigue crack propagation $\Delta K_{\mathrm{th}}$ is of paramount importance in fracture mechanics based fatigue assessment procedures. The standards ASTM E647 and ISO 12108 introduce operational definitions of $\Delta K_{\mathrm{th}}$ based on the crack propagation rate da/dN and suggest linear fits of logarithmic ΔK– da/dN test data to calculate $\Delta K_{\mathrm{th}}$. Since these fits typically suffer from a poor representation of the actual curvature of the crack propagation curve, a method for evaluating $\Delta K_{\mathrm{th}}$ using a nonlinear function is proposed. It is shown that the proposed method reduces the artificial conservativeness induced by the evaluation method as well as the susceptibility to scatter in test data and the influence of test data density.

## 1. Procedures for the Determination of the Fatigue Crack Propagation Threshold from Crack Propagation Data

Typically, the outcome of fatigue crack growth (FCG) tests for the determination of the fatigue crack propagation threshold $\Delta K_{\mathrm{th}}$ are the crack length *a* and load history data (e.g., minimum and maximum force) on dependence of the number of cycles *N*. Considering linear elastic fracture mechanics, the stress intensity factor range ΔK can be calculated from the load and crack length history [1,2,3]. By using, e.g., the numerical differentiation technique like the secant method or the incremental polynomial method [4], fatigue crack propagation rate da/dN can be computed from the crack length readings and the cycles count. Due to measurement inaccuracy, influence of the testing environment, material inhomogeneities and other effects, test data are always affected by scatter. Furthermore, in most cases, there is no distinct reading at $da/dN=da/dN_{\mathrm{th},\mathrm{ASTM}}$ and $da/dN=da/dN_{\mathrm{th},\mathrm{ISO}}$, respectively. Hence, the direct determination of the corresponding stress intensity ranges $\Delta K_{\mathrm{th},\mathrm{ASTM}}$ and $\Delta K_{\mathrm{th},\mathrm{ISO}}$ is not possible. Therefore, data fitting including inter- or extrapolation techniques to determine $\Delta K_{\mathrm{th},\mathrm{ASTM}}$ and $\Delta K_{\mathrm{th},\mathrm{ISO}}$ are needed, which will be discussed in the following.

Especially in the presence of extrinsic effects, i.e., e.g., crack closure effects [5], the accurate determination of the fatigue crack propagation threshold $\Delta K_{\mathrm{th}}$ is not trivial. The goal of this contribution is to investigate different methods for the evaluation of the fatigue crack propagation threshold and discuss their robustness and conservativeness.

Following the two most well-known standards regarding FCG tests, namely ASTM E647 [4] and ISO 12108 [6], $\Delta K_{\mathrm{th}}$ is defined as the asymptotic value of stress intensity factor range ΔK at which the fatigue crack propagation rate da/dN approaches zero. The technical (or also operational) definition of $\Delta K_{\mathrm{th}}$ for most materials is given at finite crack growth rates $da/dN_{\mathrm{th},\mathrm{ASTM}}=10^{-7}\;\mathrm{mm}/\mathrm{cycle}$ according to ASTM and $da/dN_{\mathrm{th},\mathrm{ISO}}=10^{-8}\;\mathrm{mm}/\mathrm{cycle}$ according to ISO. Since neither the fatigue crack growth rate nor the fatigue crack growth threshold stress intensity factor range can be measured directly, the data evaluation is of paramount importance.

### 1.1. Procedure Suggested by Both ASTM and ISO Standards

**Figure 1:**
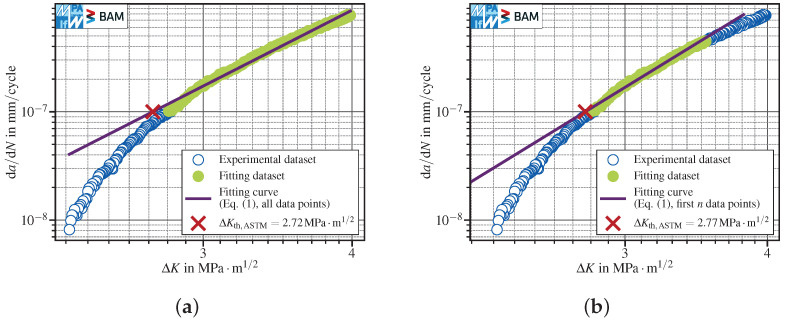
FCG test conducted with conventional *K*-decreasing at R=0.8 in lab air, see corresponding paragraph in Section 3.1.1: (**a**) linear fit over all data points with $10^{-7}\;\mathrm{mm}/\mathrm{cycle}\leq da/dN\leq 10^{-6}\mathrm{mm}/\mathrm{cycle}$; (**b**) best linear fit over the first *n* data points with $da/dN\geq 10^{-7}\;\mathrm{mm}/\mathrm{cycle}$, where n=112 (out of 177 within the interval) is selected yielding the highest Pearson correlation coefficient.

According to ASTM and ISO standards, the threshold stress intensity factor ranges are evaluated by determining ‘the best fit straight line’ [4,6] to logΔK–logda/dN data,

(1) $$\log_{10}\Delta K=P_{1}\cdot \log_{10}da/dN+P_{0},$$

where $P_{0},P_{1}$ are fitting parameters, and then calculating the stress intensity ranges corresponding to $da/dN_{\mathrm{th},\mathrm{ASTM}}$ and $da/dN_{\mathrm{th},\mathrm{ISO}}$, respectively. Both standards define a minimum number of five data points, approximately equally spaced in da/dN. The fitting interval includes data pairs between $10^{-7}\;\mathrm{mm}/\mathrm{cycle}$ and $10^{-6}\;\mathrm{mm}/\mathrm{cycle}$ for ASTM and between $10^{-8}\;\mathrm{mm}/\mathrm{cycle}$ and $10^{-7}\;\mathrm{mm}/\mathrm{cycle}$ for ISO, even though both standards allow for using additional data with lower fatigue crack propagation rates, but require documenting the modified range within the test protocol. Since nowadays FCG tests typically yield far more than five data points within one decade of da/dN data, there is plenty of room for interpretation of the suggested methods. Probably, the most straightforward interpretation (named “interpretation one” in the following) is to just take all data points within the specified ranges (as long as they are approximately equally distributed in da/dN direction) and then identify the best fit straight line for example by utilizing the least-squares method; see Figure 1a. Thereby, Equation (1) is fitted to the (logarithmic) test data, using ΔK as the dependent variable (i.e., the direction of the estimated error). The optimal set of parameters obtained by the least-squares parameter optimization returns the “best fit”. Another interpretation (named “interpretation two” in the following) might be that one may freely select n≥5 approximately equally spaced points that lie within the defined boundaries, for example starting with the point next to the desired threshold fatigue crack propagation rate. Then, the fit showing the maximum Pearson correlation coefficient is selected; see Figure 1b. The results differ and, by adding data generated at lower decades of da/dN, even a non-conservative (= higher) FCG threshold stress intensity range might be calculated, see Section 3.1.3. Furthermore, neither of both interpretations (nor any other straight line) is able to reflect the curvature of the depicted test data.

The results obtained following interpretation are strongly affected by the test data curvature because of its averaging character. The higher the curvature, the more conservative is the calculated $\Delta K_{\mathrm{th}}$. In contrast, interpretation two is less affected by data curvature because of its definition using the correlation coefficient of the straight line. In case of a more pronounced scatter in the vicinity of $da/dN_{\mathrm{th}}$, interpretation two would have accounted for a higher number of data points and therefore be more conservative. It is trivial to state that a straight line is unable to characterize the curvature of the crack growth curve and therefore leads to conservative $\Delta K_{\mathrm{th}}$ results for strictly monotonic increasing FCG data fitted within the recommended ranges. In turn, the evaluation error depends on the degree of the curvature, and it is therefore sensitive to the number (and location) of data pairs taken into account as well as to the curve’s gradient, see also [7]. Therefore—as also mentioned in the ASTM standard [4]—a nonlinear relationship between ΔK and da/dN might be beneficial to obtain a good fit.

### 1.2. Procedures Suggested in the Literature

**Figure 2:**
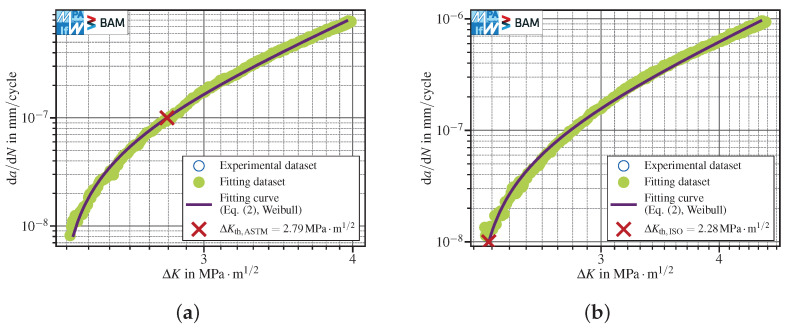
Application of a four-parameter Weibull function on all test data, referring to: (**a**) a threshold definition at $da/dN_{\mathrm{th},\mathrm{ASTM}}=10^{-7}\;\mathrm{mm}/\mathrm{cycle}$. *K*-decreasing test at R=0.8 in lab air, see corresponding paragraph in Section 3.1.1; (**b**) a slightly non-conservative FCG threshold determination at $da/dN_{\mathrm{th},\mathrm{ISO}}=10^{-8}\;\mathrm{mm}/\mathrm{cycle}$. $K_{\max}=\mathrm{const}.$, $R_{\max}\approx 0.8$, lab air, see corresponding paragraph in Section 3.1.1.

Bucci [7] considered a four-parameter Weibull function (see also [8,9]), fitted to the entire data set, see Figure 2a. He stated that the Weibull approach addresses nonlinearity in a better way, but if there are enough data points in the near-threshold region, straight line fits are performing quite well and are easier to use. In Figure 2a, the test data have been fitted using the four-parameter Weibull function

(2) $$1-\frac{\Delta K}{K_{b}}=\exp\left(-{\left(\frac{da/dN-e}{v-e}\right)}^{k}\right)$$

and $\Delta K_{\mathrm{th},\mathrm{ASTM}}$ has been determined at $da/dN_{\mathrm{th},\mathrm{ASTM}}$. Smith and Hoeppner [9] observed that the least-squares method is not suitable to fit the instability parameter $K_{b}$, the threshold parameter *e*, the characteristic value *v* and the shape parameter *k* to da/dN–ΔK data accurately within the threshold region due to the differences in orders of magnitude of the non-logarithmic test data (da/dN ranges between $10^{-7}\;\mathrm{mm}/\mathrm{cycle}$ and $10^{-6}\;\mathrm{mm}/\mathrm{cycle}$ vs. ΔK ranges between $10^{0}\;\mathrm{MPa}\cdot m^{1/2}$ and $10^{1}\;\mathrm{MPa}\cdot m^{1/2}$, approximately). Therefore, they proposed to use a least-squares optimization to calculate a preliminary optimized parameter set and afterwards improve the fitting results by optimizing only *e* and *v* in an orthogonal distance regression (ODR) [10]. Since an ODR optimization is connected to a quite high computational cost and compared to the 1990s the computation power increased dramatically until nowadays, this might have been the reason why in [9] only the two parameters showing the major contribution with regard to the mentioned errors were optimized using an ODR. In fact, it has been observed in this work that the curve fit may be improved by optimizing all four parameters instead of just two in an ODR after least-squares minimization. As one can clearly see, the overall agreement to the test data is quite good but locally diverges slightly. This can be observed in Figure 2a as well as in Figure 2b, where the four-parameter Weibull fit predicts a too steep curvature towards the ISO operational threshold definition ($da/dN_{\mathrm{th}}=10^{-8}\;\mathrm{mm}/\mathrm{cycle}$) and therefore leads to non-conservative results for $\Delta K_{\mathrm{th},\mathrm{ISO}}$. Since non-conservative values are not acceptable, the Weibull fit has not been considered any further.

**Figure 3:**
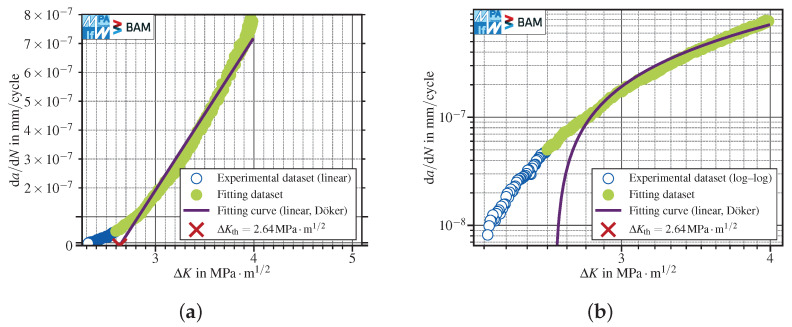
FCG threshold determination using a linear fit, applied to regular (non-logarithmic) test data, following [11]. *K*-decreasing test at R=0.8 in lab air, see corresponding paragraph in Section 3.1.1. (**a**) linear-scaled data; (**b**) double-logarithmic scaled data. The method clearly provides a poor data fit and determination of $\Delta K_{\mathrm{th}}$ at $da/dN_{\mathrm{th}}=0$.

Another well established method to evaluate the threshold stress intensity range was proposed by Döker [11]. The method uses a straight line fit applied to the (non-logarithmic) da/dN–ΔK data in the range $5\times 10^{-8}\mathrm{mm}/\mathrm{cycle}\leq da/dN\leq 10^{-6}\;\mathrm{mm}/\mathrm{cycle}$, see Figure 3a. The threshold definition is independent from the ASTM or ISO standards, and $\Delta K_{\mathrm{th}}$ is evaluated at $da/dN_{\mathrm{th}}=0$. The resulting threshold stress intensity factor range is located somewhere between the values one would expect using the ISO or ASTM operational definitions. Considering that only the ASTM E647 standard [12] is cited within the original publication, this method shows quite conservative results using the ASTM operational definition of $\Delta K_{\mathrm{th}}$. The straightforward extension to the ISO threshold by shifting the fit range by one decade of da/dN would require test results as low as $da/dN=5\times 10^{-9}\;\mathrm{mm}/\mathrm{cycle}$, which would be very time consuming to obtain and therefore not practicable. The curve behavior is poorly described, both for linear, Figure 3a, and double-logarithmic, Figure 3b, scaled da/dN–ΔK data. Therefore, further analyses with this approach have also been discarded.

Furthermore, there is a multitude of different crack propagation laws aiming at describing the whole FCG data starting from the threshold regime over the range where the FCG curve grows linear in a double-logarithmic scaled plot (also known as Paris regime) and some even include the region of instable crack growth; see [13,14,15,16,17,18,19]. Several of these models contain $\Delta K_{\mathrm{th}}$ as a model parameter, which should not be confused with the operational definitions of the threshold stress intensity factor range included in the standards.

## 2. Experimental Procedure

Since none of the methods shown in Section 1.1 and Section 1.2 allowed universal application for both standards and simultaneously led to robust and not overly conservative results, further fitting functions have been investigated. A comprehensive set of fatigue crack growth data recorded at BAM Berlin and MPA-IfW Darmstadt has been used to calibrate and validate the evaluation procedure. The fitting has been performed using least squares minimization, where the threshold stress intensity factor range has been used as dependent variable, if not otherwise mentioned.

### 2.1. Investigated Fitting Functions

The most straightforward approach was to extend Equation (1) to a more general polynomial form as

(3) $$\log_{10}\Delta K=\sum_{i=0}^{I}P_{i}\cdot {\left(\log_{10}da/dN\right)}^{i},$$

where *I* denotes the polynomial degree and $P_{i}$ are fit parameters. For I=1, Equation (3) equals Equation (1). Regarding I≥2, in case there are only very few (or even no) data points available in the vicinity of $da/dN_{\mathrm{th}}$, the fit may yield a curve having an inflection point. Hence, the fit does not represent the data in a satisfactory manner, and (for I=2) it is even possible that the fit does not intersect the $da/dN_{\mathrm{th}}$ line. Therefore, it is not recommended to use polynomial fits of the type given in Equation (3) with I≠1. The use of non-logarithmic data, like in the approach of Döker [11], leads to the same problems. Hence, further approaches have been investigated.

**Figure 4:**
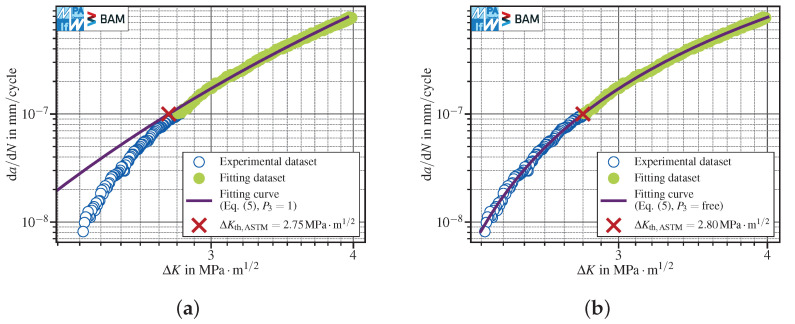

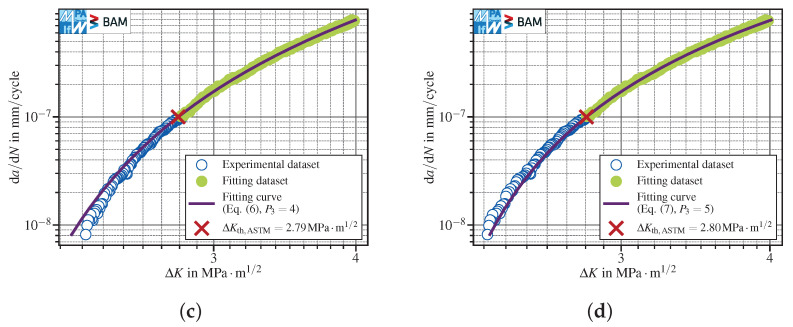
FCG threshold evaluation according to ASTM ($da/dN_{\mathrm{th},\mathrm{ASTM}}=10^{-7}\;\mathrm{mm}/\mathrm{cycle}$) for the data set presented in Figure 1 using Equation (5) with fixed or free parameter $P_{3}$: (**a**) fixed, $P_{3}=1$; (**b**) free, optimized value $P_{3}\approx 4.60$; (**c**) fixed, $P_{3}=4$; (**d**) fixed, $P_{3}=5$.

The log−log data depicted in Figure 1 considered swapping the axes, suggesting a hyperbolic trend, leading to the fit function

(4) $$\log_{10}\Delta K=P_{1}\cdot {\left(-\log_{10}da/dN\right)}^{-1}+P_{2},$$

with the fitting parameters $P_{1}$ and $P_{2}$. Fitting Equation (4) to the dataset leads to results comparable to the linear fit using all data points in accordance with the standards, since the exponent −1 does not represent the curvature of the crack growth curve, see Figure 4a. By extending Equation (4) to a variable exponent as

(5) $$\log_{10}\Delta K=P_{1}\cdot {\left(-\log_{10}da/dN\right)}^{-P_{3}}+P_{2},$$

with $P_{3}\geq 1$, a much better fitting to the test data is possible, see Figure 4b. Rounding the fit result of $P_{3}\approx 4.60$ to the previous and next integer number, namely $P_{3}=4$,

(6) $$\log_{10}\Delta K=P_{1}\cdot {\left(-\log_{10}da/dN\right)}^{-4}+P_{2},$$

see Figure 4c and $P_{3}=5$,

(7) $$\log_{10}\Delta K=P_{1}\cdot {\left(-\log_{10}da/dN\right)}^{-5}+P_{2},$$

see Figure 4d provides two straightforward variants of Equation (5) with only two free parameters. The curvature of the fitting curve increases with increasing $P_{3}$. It is worth noting that, although $P_{3}=5$ leads to non-conservative estimation of the FCG threshold according to ISO, it is still conservative following the ASTM operational definition.

### 2.2. Quantitative Data Analysis

**Figure 5:**
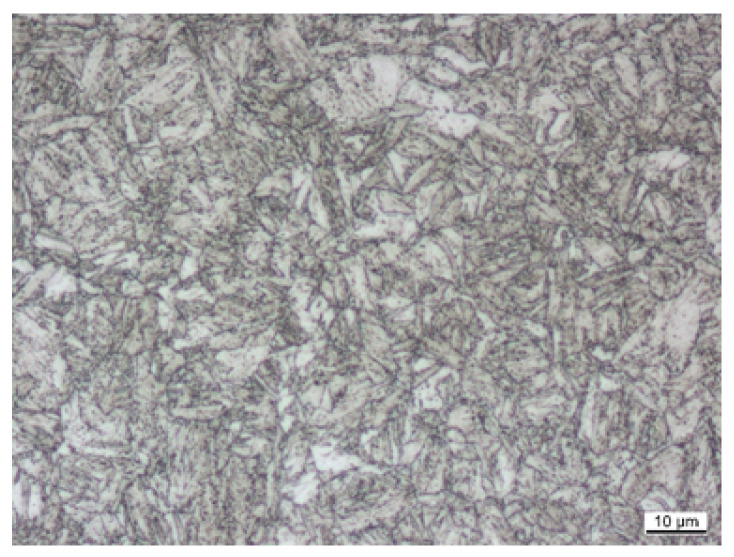
S690QL microstructure (T–L plane).

The main datasets investigated stemmed from a total of 47 specimens manufactured from 12mm thick S690QL hot-rolled plates, tested at BAM Berlin and MPA-IfW Darmstadt. The materials chemical composition and mechanical properties are given in Table 1 and Table 2. The microscopic analysis on etched samples showed a fine grained quenched and tempered martensitic-bainitic microstructure, see Figure 5.

All tests were performed using SENB specimens [6] with a cross section of 19mm×6mm. The specimens were oriented, such that the direction of crack propagation is parallel to the rolling direction, i.e., the orientation T–L according to [20]. The test data have been obtained on three resonance testing machines equipped with an eight-point bending fixture, see [6]. These were a RUMUL MIKROTRON 654 with a maximum load capacity of 20kN and an average testing frequency of about 108Hz, a RUMUL TESTRONIC with a maximum load capacity of 100kN and an average testing frequency of about 60Hz, both at BAM Berlin, and a RUMUL TESTRONIC with a maximum load capacity of 250kN and an average testing frequency of about 90Hz at MPA-IfW Darmstadt. The crack length was monitored using direct current potential drop techniques with current reversal and active temperature compensation (current source: HP 6033A, nanovolt meter: Keithley 2182A) at BAM and specimen compliance techniques (clip-gage: Sandner EXR10-0.5o) at MPA-IfW. The crack length was corrected a posteriori by means of optical measurements on the broken open fracture surfaces. Then, the crack propagation rates have been calculated using the slope of piecewise straight line fits performed on filtered test data. Each segment of the piecewise function referred to a crack extension of 0.02mm. The corresponding stress intensity factors have been calculated using the formulations reported in [6].

Since in these tests the same specimen types, manufactured from the same material batch in the same specimen orientation, have been used, a high repeatability was expected. Consequently, the standard deviation in $\Delta K_{\mathrm{th}}$ calculated for each method was the result of the data scatter within the test (stemming from small variations in environment conditions, material inhomogeneities, specimen misalignment, errors in the calculation of da/dN, etc.) and an error induced by the fit used to evaluate $\Delta K_{\mathrm{th}}$. Since the first part is independent of the fitting procedure, the differences in standard deviations are a measure for the fit robustness, whereas the corresponding mean value gives information on the fit quality and therefore the inter- and extrapolation error, respectively.

## 3. Results and Discussion

### 3.1. Application to Data Obtained at R≈0.8

First, data obtained at a load ratio of approximately R≈0.8, which produce only a negligible influence of crack closure effects have been investigated.

#### 3.1.1. Evaluation for the Intervals Suggested by the Standards

In order to assess the performance of the polynomial functions with negative exponent Equation (5) in comparison to the fit suggested by the standards, see Equation (1), test data obtained at either a fixed load ratio R=0.8 (*K*-decreasing procedures) or at $R_{\max}\approx 0.8$ ($K_{\max}$ tests) have been considered, see ([4], Section 8.6). To minimize influences of the extrapolation method, the smallest recorded crack propagation rate has been required to be smaller than $1.1\cdot da/dN_{\mathrm{th}}$, i.e., $\min\left(da/dN\right)\leq 1.1\times 10^{-8}\;\mathrm{mm}/\mathrm{cycle}$ for the ISO and $\min\left(da/dN\right)\leq 1.1\times 10^{-7}\;\mathrm{mm}/\mathrm{cycle}$ for the ASTM operational definition of $\Delta K_{\mathrm{th}}$. It shall be noted that these boundaries have been used only for comparability between tests within this work and neither define the actual application boundaries of the method regarding data extrapolation nor represent a general recommendation.

Since the fixed parameters $P_{3}=4$ in Equation (6) and $P_{3}=5$ in Equation (7) provided a better description of the data curvature and a less conservative determination of $\Delta K_{\mathrm{th}}$ in addition to the more general function with variable $P_{3}$, these have been compared with the two interpretations of the linear fit method suggested by the standards, see Figure 1a,b. The range used to fit the data was in all cases fixed to one decade of da/dN data, starting from $da/dN_{\mathrm{th}}$ and therefore equal to the ranges suggested by the standards in order to ensure comparability between the methods.

##### Conventional *K*-Decreasing at R=0.8 ($\Delta K_{\mathrm{LR}}$)

The first datasets investigated stemmed from a total of nine SENB specimens made of S690QL. These tests have been conducted using the standard *K*-decreasing (or load shedding) procedure suggested by the standards at constant R=0.8, tested in lab air. Further information on the experimental procedure may be found in [21]. All nine specimens contained data points below $da/dN=1.1\times 10^{-7}\;\mathrm{mm}/\mathrm{cycle}$ and are valid for ASTM operational threshold evaluation, and four of them were also valid according to the ISO definition. The threshold stress intensity factor range results with corresponding standard deviations are presented in Table 3.

The comparison between the determined threshold stress intensity factor ranges (on single specimen basis) and the value read out from test data proved conservative for every single method and dataset. Considering the mean value of the threshold stress intensity ranges, both linear fits showed a more pronounced underestimation of $\Delta K_{\mathrm{th}}$, inducing an artificial conservativeness, as already observed in Section 2.1. This is proven true especially for the linear fit Equation (1) performed on all data points. For the linear fit over the first *n* points, the artificial conservativeness of $\Delta K_{\mathrm{th},\mathrm{ASTM}}$ was found to be comparatively higher than for $\Delta K_{\mathrm{th},\mathrm{ISO}}$. The results obtained using Equation (5) agreed fairly well, whereas the fixed exponents $P_{3}=4$ and $P_{3}=5$ showed a slightly higher conservativeness for $\Delta K_{\mathrm{th},\mathrm{ISO}}$ compared to the three-parameter version of Equation (5). The standard deviation is very low and comparable for all tests.

##### Load Shedding at Constant $K_{\max}$ ($R_{\max}\approx 0.8$)

A set of nine SENB prepared from the same material batch has been investigated using a load shedding scheme at constant $K_{\max}$ with a final load ratio $R_{\max}\approx 0.8$ at about $\Delta K_{\mathrm{th},\mathrm{ISO}}$ and R≈0.72…0.76 at $\Delta K_{\mathrm{th},\mathrm{ASTM}}$. All nine specimens provided data for evaluating $\Delta K_{\mathrm{th},\mathrm{ASTM}}$ and among them four were also valid for $\Delta K_{\mathrm{th},\mathrm{ISO}}$ evaluation, see Table 4.

Since the crack propagation data obtained from different procedures at R≈0.8 nearly coincide and exhibit a very low scatter, the same conclusion as in the case of conventional *K*-decreasing tests can be drawn.

##### Compression Precracking Load Reduction (CPLR) at R=0.8

A further set of four SENB extracted from the same batch has been investigated using compression precracking followed by a *K*-decreasing test with a constant load ratio R=0.8. Applying the same criteria for selecting valid data sets for comparison returned four specimens for evaluating $\Delta K_{\mathrm{th},\mathrm{ASTM}}$ and two for $\Delta K_{\mathrm{th},\mathrm{ISO}}$, see Table 5. Here, the standard deviation for ISO is omitted due to the insufficient number of available data sets that include points below $da/dN=1.1\times 10^{-8}\;\mathrm{mm}/\mathrm{cycle}$. The results confirm those previously shown in Table 4.

##### Constant Force Range (ΔF-Constant) at R=0.8

The fourth and last test to determine the threshold at R≈0.8 in ambient air has been based on another set of four SENB specimens produced from the same material batch, using conventional precracking followed by a test at constant force amplitude (ΔF-constant) at a load ratio R=0.8. All four specimens have been considered valid for evaluating $\Delta K_{\mathrm{th},\mathrm{ASTM}}$ and three for $\Delta K_{\mathrm{th},\mathrm{ISO}}$, see Table 6. Here, the same observations as above apply.

##### Summary

The linear fits induce artificial conservativeness in the evaluation of fatigue crack propagation thresholds obtained at R≈0.8. Nevertheless, this behavior is observed less pronounced for the linear fit incorporating only the first *n* points. In all cases, the three-parameter polynomial Equation (5) provides less conservative results. Fixing its parameter $P_{3}$ to $P_{3}=4$ or $P_{3}=5$ sometimes induces conservativeness, but in most of the cases less pronounced than the linear fits.

**Figure 6:**
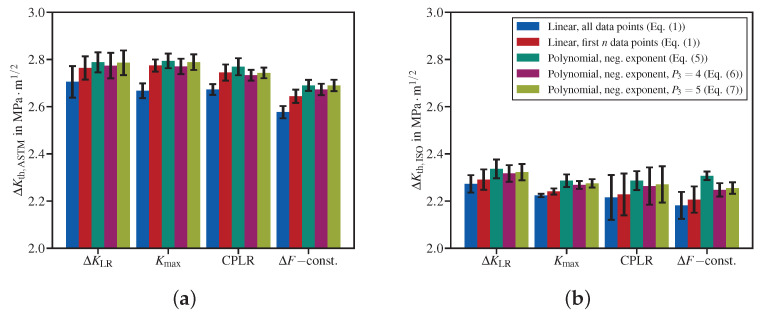
Fatigue crack propagation thresholds obtained at R≈0.8 applying the fit methods to the four datasets presented in Section 3.1.1: (**a**) according to the ASTM operational definition; (**b**) according to the ISO operational definition.

Figure 6 summarizes the evaluation of the four datasets presented earlier in Section 3.1.1. The threshold stress intensity factor ranges determined according to the ASTM operational definition (see Figure 6a) as well as those following the ISO operational definition (see Figure 6b) for the four test methods ($\Delta K_{\mathrm{LR}}$, $K_{\max}$, CPLR and ΔF-constant) agree fairly well within each data evaluation method.

#### 3.1.2. Robustness of the Fitting Methods in Handling Data Subjected to Augmented Artificial Scatter

In order to assess the ability and robustness of the fitting methods to handle scattered data, artificial scatter has been added to the test data presented in Figure 1. The additional scatter has been generated by sampling random values from a normal distribution with a mean μ=1 and a standard deviation SD=0.02 and multiplying them with ΔK data, whereas da/dN remained unchanged.

**Figure 7:**
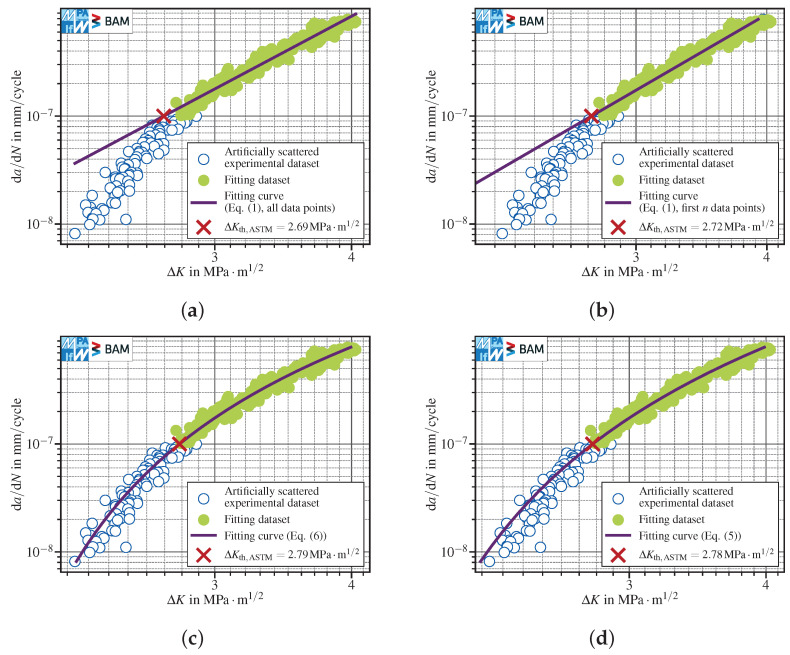
FCG data reported in Figure 1 with additional artificial scatter. The $da/dN_{\mathrm{th},\mathrm{ASTM}}$ has been evaluated using four different methods: (**a**) linear, all data points Equation (1); (**b**) linear, first n=176 data points (out of 177 within the interval) Equation (1); (**c**) polynomial, negative exponent, $P_{3}=4$ Equation (6); (**d**) polynomial, negative exponent Equation (5).

The comparison of the linear fit using all scattered data points within the defined interval ($\Delta K_{\mathrm{th},\mathrm{ASTM}}=2.69\;\mathrm{MPa}\cdot m^{1/2}$, Figure 7a) with the original dataset ($\Delta K_{\mathrm{th},\mathrm{ASTM}}=2.72\;\mathrm{MPa}\cdot m^{1/2}$, Figure 1a) did not reveal a notable difference. The polynomials with negative exponents were also almost insensitive to scatter. The three-parameter polynomial provided $\Delta K_{\mathrm{th},\mathrm{ASTM}}=2.78\;\mathrm{MPa}\cdot m^{1/2}$ for the scattered data (Figure 7d) compared to $\Delta K_{\mathrm{th},\mathrm{ASTM}}=2.80\;\mathrm{MPa}\cdot m^{1/2}$ for the original dataset (Figure 4b). The two-parameter polynomial with $P_{3}=4$ showed a similar trend with $\Delta K_{\mathrm{th},\mathrm{ASTM}}=2.79\;\mathrm{MPa}\cdot m^{1/2}$ for scattered data (Figure 7c) in comparison to $\Delta K_{\mathrm{th},\mathrm{ASTM}}=2.79\;\mathrm{MPa}\cdot m^{1/2}$ for the original dataset (Figure 4c).

In contrast, the best linear fit over the first *n* points showed a pronounced sensitivity to scattered data. The best fit interval coincided almost with all data points (see Figure 7b and Figure 1b). Consequently, $\Delta K_{\mathrm{th},\mathrm{ASTM}}=2.72\;\mathrm{MPa}\cdot m^{1/2}$ calculated for scattered data was more conservative than $\Delta K_{\mathrm{th},\mathrm{ASTM}}=2.77\;\mathrm{MPa}\cdot m^{1/2}$ calculated in case of the original dataset.

#### 3.1.3. Influence of an Augmented Fit Interval

**Figure 8:**
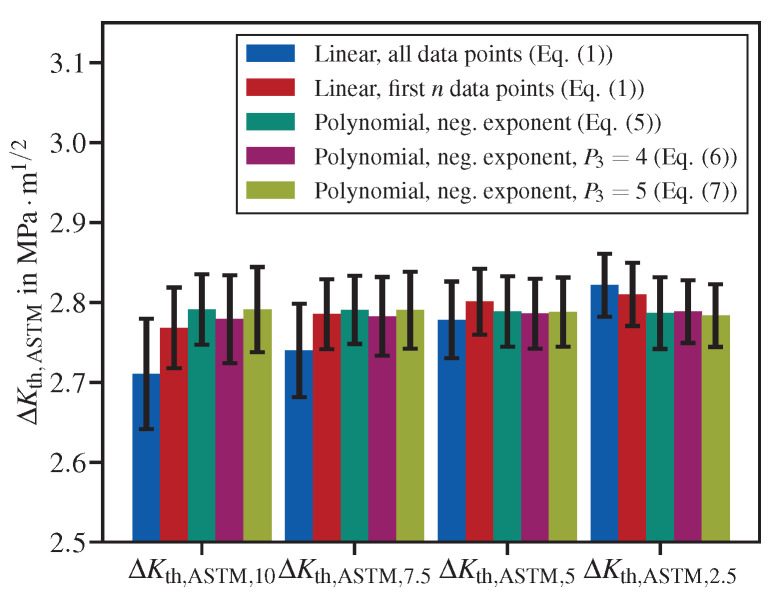
Comparison of threshold values obtained with various fit methods using varying fit intervals from $2.5\times 10^{-8}\;\mathrm{mm}/\mathrm{cycle}\leq da/dN\leq 10^{-6}\;\mathrm{mm}/\mathrm{cycle}$ ($\Delta K_{\mathrm{th},\mathrm{ASTM},2.5}$) up to $10\times 10^{-8}\;\mathrm{mm}/\mathrm{cycle}\leq da/dN\leq 10^{-6}\;\mathrm{mm}/\mathrm{cycle}$ ($\Delta K_{\mathrm{th},\mathrm{ASTM},10}$). The results refer to eight specimens made of S690QL, tested using a *K*-decreasing procedure at R=0.8 in lab air.

Both ASTM E647 and ISO 12108 suggest a fit interval of one decade of da/dN data, starting from $da/dN_{\mathrm{th},\mathrm{ASTM}}$ and $da/dN_{\mathrm{th},\mathrm{ISO}}$, respectively. Nevertheless, both allow for use data obtained at lower fatigue crack propagation rates for determining the threshold stress intensity factor range. Therefore, the impact of an augmented fit interval on the determination of fatigue crack propagation thresholds has been investigated. Since no datasets with crack propagation rates momentously below $da/dN_{\mathrm{th},\mathrm{ISO}}=1\times 10^{-8}\;\mathrm{mm}/\mathrm{cycle}$ were available, only the threshold following the ASTM operational definition has been considered. Therefore, the data shown in Table 3, which have been obtained using a fit interval of $10^{-7}\;\mathrm{mm}/\mathrm{cycle}\leq da/dN\leq 10^{-6}\;\mathrm{mm}/\mathrm{cycle}$, have been compared with threshold stress intensity factor ranges obtained with augmented intervals. The upper bound has been held constant, whereas the lower bound has been varied from $2.5\times 10^{-8}\;\mathrm{mm}/\mathrm{cycle}$ up to $10^{-7}\;\mathrm{mm}/\mathrm{cycle}$. The respective threshold stress intensity factor ranges have been named as $\Delta K_{\mathrm{th},\mathrm{ASTM},2.5}$, $\Delta K_{\mathrm{th},\mathrm{ASTM},5}$, $\Delta K_{\mathrm{th},\mathrm{ASTM},7.5}$ and $\Delta K_{\mathrm{th},\mathrm{ASTM},10}$, according to the lower FCG propagation rate bounds ($2.5,\;5,\;7.5\;\mathrm{and}\;10\times 10^{-8}\;\mathrm{mm}/\mathrm{cycle}$, see Figure 8).

Regarding both linear fits, there is a clear tendency that, with augmenting the fit interval towards lower minimal crack propagation rates (displayed in Figure 8 from left to right), there is an increase in $\Delta K_{\mathrm{th},\mathrm{ASTM}}$ and therefore a decrease in conservativeness. In contrast, the results for the polynomial with negative exponent, Equations (5)–(7), are almost insensitive to the interval augmentation, but for $P_{3}=4$ and $P_{3}=5$, a reduction in standard deviation can be observed with increasing the interval. For Equation (6), the optimal lower bound was found at $2.5\times 10^{-8}\;\mathrm{mm}/\mathrm{cycle}$ and $5\times 10^{-8}\;\mathrm{mm}/\mathrm{cycle}$ with equal magnitudes in mean and standard deviation.

**Figure 9:**
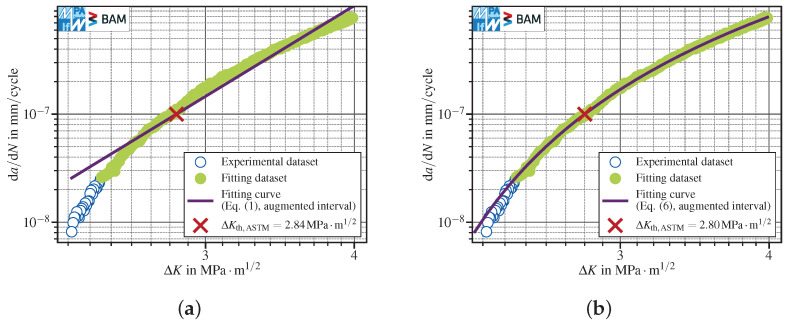
Influence of augmented fit interval on various fits, using an interval $2.5\times 10^{-8}\;\mathrm{mm}/\mathrm{cycle}\leq da/dN\leq 10^{-6}\;\mathrm{mm}/\mathrm{cycle}$: (**a**) the linear fit using all data within the specified range leads to a non-conservative result; (**b**) the polynomial fit with negative exponent $P_{3}=4$ gives a conservative result. The same dataset as reported in Figure 1 has been used.

Using the linear functions with augmented intervals increases the risk of non-conser-vative extrapolation, as one can see comparing the values for $\Delta K_{\mathrm{th},\mathrm{ASTM},2.5}$, where the linear functions provided the highest threshold stress intensity factor ranges among all five methods under comparison. This issue can be clearly understood looking at the evaluation depicted in Figure 9. In particular, Figure 9a shows that the $\Delta K_{\mathrm{th},\mathrm{ASTM}}$ calculated using the linear fit over all data points is on the right-hand side of the dataset, i.e., in the non-conservative region. In contrast, the polynomial with $P_{3}=4$ does not show this issue (Figure 9b).

#### 3.1.4. Data Extrapolation

The investigations presented in Section 3.1.1 included only data at crack propagation rates as low as $1.1\cdot da/dN_{\mathrm{th}}$. Nevertheless, it shall be noted that no data might be available at low crack propagation rates, especially for the ISO operational definition with a threshold crack propagation rate as low as $da/dN_{\mathrm{th},\mathrm{ISO}}=10^{-8}\;\mathrm{mm}/\mathrm{cycle}$. Therefore, to ensure a reliable and conservative evaluation of the fatigue crack propagation thresholds, a robust extrapolation technique is needed. To assess the goodness of the extrapolation, the test results given in Section 3.1.1 have been compared to artificially censored datasets, using only data with $da/dN\geq 3\cdot da/dN_{\mathrm{th}}$. Hence, the resulting fit intervals after censoring were $3\times 10^{-8}\;\mathrm{mm}/\mathrm{cycle}\leq da/dN\leq 10^{-7}\;\mathrm{mm}/\mathrm{cycle}$ for ISO operational definition and $3\times 10^{-7}\mathrm{mm}/\mathrm{cycle}\;\leq da/dN\leq 10^{-6}\;\mathrm{mm}/\mathrm{cycle}$ for ASTM operational definition. Since extrapolation is very sensitive to the data range available, in order to have a reliable comparison, the investigations have been restricted to datasets that had data within the whole censored interval (including the upper bound). Hence, the number of tests with valid data for ASTM threshold determination is reduced in comparison to Section 3.1.1.

The comparison has been based on the change in $\Delta K_{\mathrm{th}}$ induced by censoring the FCG data. $\Delta K_{\mathrm{th},\mathrm{cens}}$ denotes the fatigue crack propagation threshold obtained for the censored version of the data set used to evaluate $\Delta K_{\mathrm{th}}$. It follows that $\Delta K_{\mathrm{th}}-\Delta K_{\mathrm{th},\mathrm{cens}}$ values greater than or equal to zero are considered conservative, whereas values lower than zero are non-conservative. The minimum difference throughout all specimens shows whether all tests are extrapolated conservatively, whereas the mean value can be regarded as an index of the goodness of the extrapolation. The results are given in Table 7. Conservative extrapolation has been obtained for both linear fits and for the polynomial with negative exponent fixed to $P_{3}=4$, whilst the versions with $P_{3}=5$ or free $P_{3}$ returned a very limited number of negative results, meaning non-conservative extrapolation results. Even though the latter are just slightly non-conservative and rare, the occurrence of a non-conservative extrapolation should be avoided whenever possible. Regarding the “$\mathrm{mean}\left(\;\cdot \;\right)$” columns, denoting the extrapolation error, the linear functions performed far worse than the polynomial Equation (5) with $P_{3}=4$, especially with regard to the $\Delta K_{\mathrm{th},\mathrm{ASTM}}-\Delta K_{\mathrm{th},\mathrm{cens},\mathrm{ASTM}}$ values, where the mean and minimum extrapolation error were $0.20\;\mathrm{MPa}\cdot m^{1/2}$ and $0.17\;\mathrm{MPa}\cdot m^{1/2}$, regarding the fit over all data points and $0.23\;\mathrm{MPa}\cdot m^{1/2}$ and $0.18\;\mathrm{MPa}\cdot m^{1/2}$ for the fit over the first *n* points, respectively, compared to $0.03\;\mathrm{MPa}\cdot m^{1/2}$ and $0.01\;\mathrm{MPa}\cdot m^{1/2}$ for the polynomial with $P_{3}=4$. Hence, in case extrapolation is inevitable, the polynomial with $P_{3}=4$ brings a notable improvement over the linear fits, suggested by the standards. With regard to both standards, neither a statement on the minimum required crack propagation rate for a valid evaluation of the FCG threshold nor any comments on the legitimacy of a potentially necessary data extrapolation is given.

#### 3.1.5. Application to the Full Dataset

Based on the conclusions drawn in Section 3.1.4, where a robust conservative extrapolation could be obtained for the linear fits as well as for the polynomial with negative exponent fixed to $P_{3}=4$, the full dataset, including all datasets that may be extrapolated, has been reevaluated. Because an augmented fit interval may lead to non-conservative results in case of linear fits, the intervals suggested in the standards have been used (see Section 1.1). In contrast, a beneficial effect of an augmented interval has been observed when using Equation (6), see Section 3.1.3. Hence, an augmented interval for calculating $\Delta K_{\mathrm{th},\mathrm{ASTM}}$ has been used in this case. Consequently, the fitting intervals have been defined as $da/dN=5\times 10^{-8}\;\mathrm{mm}/\mathrm{cycle}\leq da/dN\leq 10^{-6}\;\mathrm{mm}/\mathrm{cycle}$ for ASTM and $da/dN=10^{-8}\;\mathrm{mm}/\mathrm{cycle}\leq da/dN\leq 10^{-7}\;\mathrm{mm}/\mathrm{cycle}$ for ISO. The results are presented in Table 8. The linear fit over the full interval resulted in the highest conservativeness in combination with a small standard deviation. Using only the first *n* points of the interval to generate the linear fit reduced the conservativeness with the drawback of increasing the standard deviation, especially for ASTM. By using an appropriate nonlinear function like Equation (6), the conservativeness as well as the standard deviation can be reduced.

In order to assess the methods capabilities when dealing with an augmented scatter in ΔK, artificial scatter, as described in Section 3.1.2, is added to each specimen data set. The results are given in Table 9. Like already observed in Figure 7, the two-parameter polynomial Equation (6) is almost insensitive to scatter in test data, whereas both linear fits partially suffer from pronounced susceptibility to scattered data.

### 3.2. Definition of the Fitting Function and Interval for the Determination of Thresholds Obtained at R≈0.8

Based on the results presented in Section 3.1, we suggested to use Equation (6) for fitting the test data obtained at R≈0.8. This polynomial exhibits only a minimal dependency on scatter in test data and returns robust results that simultaneously show only a small, but persistent conservativeness. Furthermore, data extrapolation has been shown to be valid if the lowest available crack propagation rate fulfills $da/dN_{\min}\leq 3\cdot da/dN_{\mathrm{th}}$.

Since an augmented fit interval shows an additional reduction in standard deviation, we suggested to use an augmented interval of $5\times 10^{-8}\;\mathrm{mm}/\mathrm{cycle}\leq da/dN\leq 10^{-6}\;\mathrm{mm}/\mathrm{cycle}$ compared to the one proposed in the ASTM standard ($10^{-7}\;\mathrm{mm}/\mathrm{cycle}\leq da/dN\leq 10^{-6}\;\mathrm{mm}/\mathrm{cycle}$). Regarding the ISO operational definition, we recommended to use the suggested interval of $10^{-8}\;\mathrm{mm}/\mathrm{cycle}\leq da/dN\leq 10^{-7}\;\mathrm{mm}/\mathrm{cycle}$, since crack propagation rates much lower than $10^{-8}\;\mathrm{mm}/\mathrm{cycle}$ are time consuming using conventional FCG testing.

Using the herein proposed method for evaluating $\Delta K_{\mathrm{th}}$, the artificial conservativeness can be reduced and the fits’ robustness improved compared to the linear functions, as shown in the previous paragraphs. Since the evaluation following the proposed method involves a very low effort compared to the conducted experiments, the application thereof is generally preferable over the linear fits.

### 3.3. Evaluation of the Fatigue Crack Propagation Threshold at R=−1

**Figure 10:**
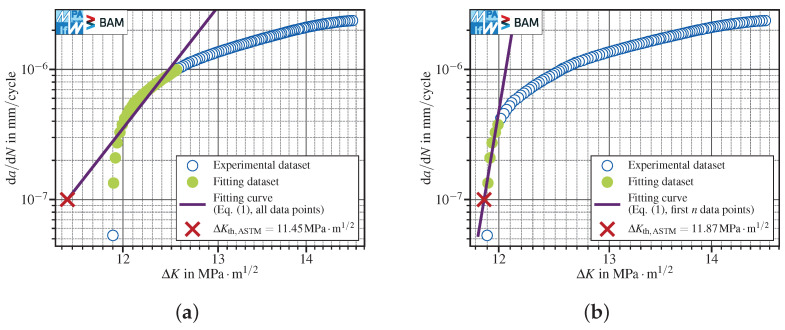

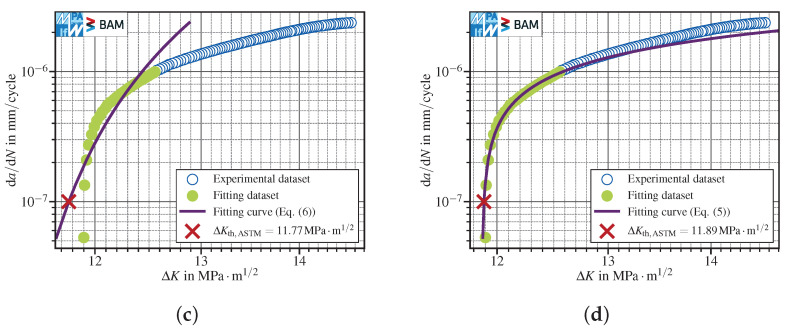
FCG data showing a distinct effect of fatigue crack closure leading to crack arrest. The tests have been conducted following a compression precracking load reduction procedure at R=−1 in lab air. Different fitting strategies have been used: (**a**) the linear fit using all data in the interval $10^{-7}\;\mathrm{mm}/\mathrm{cycle}\leq da/dN\leq 10^{-6}\;\mathrm{mm}/\mathrm{cycle}$ provided an overly conservative $\Delta K_{\mathrm{th},\mathrm{ASTM}}$ value; (**b**) the linear fit using the first n=5 data points (out of 29 within the interval) showed a fairly good $\Delta K_{\mathrm{th},\mathrm{ASTM}}$ approximation; (**c**) Equation (6), using a fixed exponent $P_{3}=4$, was not capable of reproducing the curvature and therefore gave a very conservative result; (**d**) a very good result could be achieved using the three-parameter variant of the polynomial ($P_{3}\approx 24.1$).

In contrast to tests carried out at R=0.8, specimens tested at lower load ratios like R=−1 may exhibit a distinct influence of extrinsic effects such as crack closure. This can be observed in the example reported in Figure 10: due to the progressive development of crack closure during the load shedding test, the crack propagation rate decreases rapidly in the near-threshold regime, leading to a steep crack propagation curve towards the threshold. This has major consequences on the evaluation of the fatigue crack propagation threshold due to the fact that much fewer experimental points are available in the selected fitting intervals.

The linear fit over all points within the interval (Figure 10a) is not capable of handling the pronounced curvature. Hence, $\Delta K_{\mathrm{th},\mathrm{ASTM}}$ is calculated overly conservatively. This holds true also for the polynomial with $P_{3}=4$, Equation (6) (Figure 10c). In contrast, the linear fit using the first *n* points (Figure 10b) shows a fairly good threshold approximation. The best results are obtained using the three-parameter polynomial Equation (5) (Figure 10d), which adapts to the curvature displayed by the data fairly well and results in the least (but still) conservative $\Delta K_{\mathrm{th},\mathrm{ASTM}}$ value.

#### 3.3.1. Handling of Data Affected by Extrinsic Mechanisms

**Figure 11:**
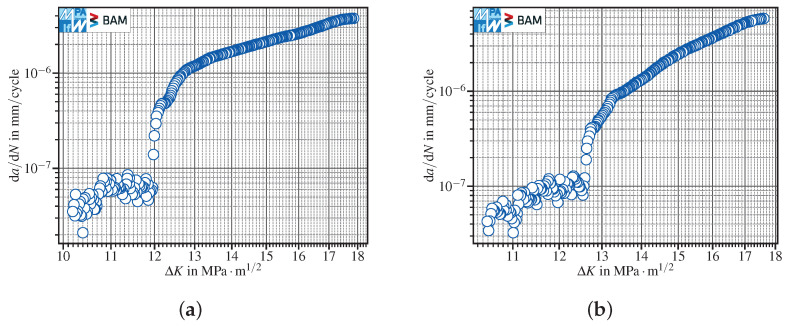
FCG data showing a kink at about the ASTM threshold ($da/dN_{\mathrm{th},\mathrm{ASTM}}=10^{-7}\;\mathrm{mm}/\mathrm{cycle}$): (**a**) kinking starts below $da/dN=10^{-7}\;\mathrm{mm}/\mathrm{cycle}$; (**b**) kinking starts slightly above $10^{-7}\;\mathrm{mm}/\mathrm{cycle}$. The data refer to conventional *K*-decreasing at R=−1 in lab air. Both tests have been interrupted, since neither crack arrest has been observed nor $da/dN_{\mathrm{th},\mathrm{ISO}}$ has been reached.

Extrinsic mechanisms affect the cyclic deformation in the crack wake and therefore influence the crack growth rate [22]. The results about tests carried out at R=0.1 and R=−1 showed a kink of the crack propagation curve in the near-threshold regime. For an in detail discussion on these findings, see [21]. The results depicted in Figure 11 about two specimens tested in lab air, using a conventional *K*-decreasing procedure at R=−1, pose the question of how to analyze the data to evaluate the fatigue crack propagation threshold. In fact, both specimens show a distinct kink in the FCG data at about $da/dN\approx 10^{-7}\;\mathrm{mm}/\mathrm{cycle}$. In such cases, the calculation of a threshold stress intensity factor range is—regardless of the standard used—questionable, since no asymptotic behavior of the da/dN-ΔK curve towards $\Delta K_{\mathrm{th}}$ is observable (see Section 1).

No general rule is applicable to the evaluation of the fatigue crack propagation thresholds, but each dataset shall be analysed separately. For instance, Figure 11a shows that the determination of $\Delta K_{\mathrm{th},\mathrm{ASTM}}$ would have been possible, since the kink started below $da/dN_{\mathrm{th},\mathrm{ASTM}}=10^{-7}\;\mathrm{mm}/\mathrm{cycle}$. If the aim of this *K*-decreasing test would have been to determine $\Delta K_{\mathrm{th},\mathrm{ASTM}}$, one might have stopped the test after reaching $da/dN<da/dN_{\mathrm{th},\mathrm{ASTM}}=10^{-7}\;\mathrm{mm}/\mathrm{cycle}$ for the first time. Hence, one would have never observed the effect of corrosion on FCG data starting just below $\Delta K\approx 12\;\mathrm{MPa}\cdot m^{1/2}$. Nevertheless, as the crack propagation data did not display a pronounced threshold behavior, a value for $\Delta K_{\mathrm{th},\mathrm{ASTM}}$ should not be provided. This holds true also for the dataset depicted in Figure 11b, in which corrosion effects started above $da/dN_{\mathrm{th},\mathrm{ASTM}}=10^{-7}\;\mathrm{mm}/\mathrm{cycle}$. In these cases, we recommend providing the last da/dN–ΔK reading recorded within the test as a pure indication of the lowest stress intensity factor range obtained in the load reduction test, which, nevertheless, must not be taken as a fatigue crack propagation threshold. Hence, an automated evaluation of FCG data should only be performed after checking the crack propagation data in the near-threshold regime.

#### 3.3.2. Influence of a Lower Data Density

**Figure 12:**
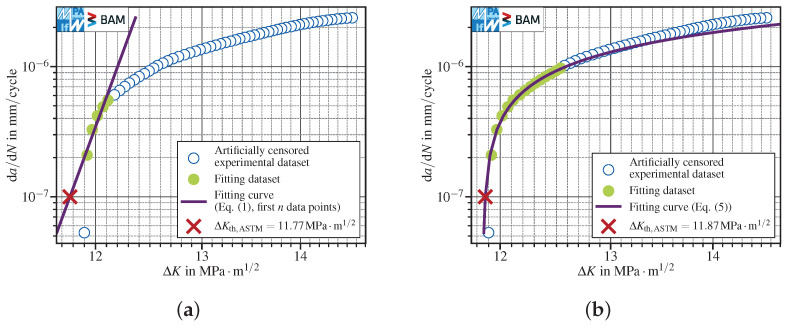
Test data presented in Figure 10 reduced by 50% to assess the influence of a lower data density next to $da/dN_{\mathrm{th},\mathrm{ASTM}}$. (**a**) the linear fit using the best first *n* data points gives an overly conservative $\Delta K_{\mathrm{th},\mathrm{ASTM}}$ value; (**b**) using the three-parameter polynomial Equation (5) shows a very good agreement with test data.

The majority of test results presented herein show very low noise in conjunction with a fairly high data density. Both properties depend on the quality of raw data and the methodology used to calculate da/dN and ΔK. In Figure 12, the test data presented in Figure 10 are reduced by a factor of two by skipping every second data point. Since still 14 data points distributed over the whole interval of $10^{-6}\;\mathrm{mm}/\mathrm{cycle}\leq da/dN\leq 10^{-7}\;\mathrm{mm}/\mathrm{cycle}$ are left, this data set clearly fulfills the requirement of providing at least five points, defined in [4].

Here, the advantages of an appropriate nonlinear fit functions apply. The linear fit using the first *n* points shows a pronounced underestimation of the threshold stress intensity range, whereas the three-parameter polynomial’s sensitivity to the number of data points is very limited.

#### 3.3.3. Validation of the Proposed Method

After examining all the possible issues which might influence the robust determination of the fatigue crack propagation threshold, the methods have been validated against various datasets.

##### Conventional *K*-Decreasing at R=−1 in Lab Air

The first datasets investigated stemmed from a total of eight SENB specimens made of S690QL, whereof three showed a kink (see Figure 11) and therefore have not been considered in the analysis. These tests have been conducted using the *K*-decreasing procedure included in the standards [4,6] at constant load ratio R=−1 in lab air. All five remaining datasets contained data points below $da/dN=1.1\times 10^{-7}\;\mathrm{mm}/\mathrm{cycle}$ and therefore have been considered valid for ASTM operational threshold evaluation, while two of them were also valid regarding the ISO definition. The evaluated fatigue crack propagation thresholds, including the corresponding standard deviations, are presented in Table 10. Note that, in case of ISO, no standard deviation has been calculated due to insufficient data available.

The conservativeness of each method has been analyzed: the two-parameter polynomials Equation (6) and Equation (7), and especially the linear fit over all points, showed a pronounced underestimation of $\Delta K_{\mathrm{th},\mathrm{ASTM}}$ in conjunction with a higher standard deviation compared to the linear fit over the first *n* points or the three-parameter polynomial Equation (5). The observations with regard to artificial conservativeness also apply to the results for $\Delta K_{\mathrm{th},\mathrm{ISO}}$. Comparing the latter two methods, both show comparable results, both regarding the mean $\Delta K_{\mathrm{th}}$ value and the standard deviation.

##### Compression Precracking Load Reduction at R=−1 in Lab Air

The datasets investigated stemmed from a total of ten SENB specimens made of S690QL. All specimens showed a distinct threshold behavior and therefore all have been considered in the analysis. These tests have been conducted using compression precracking followed by a load reduction procedure at constant R=−1 in lab air. All ten datasets contained data points below $da/dN=1.1\times 10^{-7}\;\mathrm{mm}/\mathrm{cycle}$ and therefore have been considered valid for ASTM operational threshold evaluation, whereas none of them contained points in order to calculate a threshold value according to ISO. The threshold stress intensity factor ranges with corresponding standard deviations are presented in Table 11.

The same conclusions as in the previous paragraph can be drawn: the linear fit over the first *n* points and the three-parameter polynomial Equation (5) provided the best results.

### 3.4. Definition of the Fitting Function and Interval for Tests Conducted at R≪0.8

Data obtained at load ratios momentously lower than R=0.8 might be affected by phenomena like crack-closure or corrosion, which make the determination of the fatigue crack propagation threshold difficult. When a steep gradient or a kink in the near-threshold data are observed, no general or automatic extrapolation of these test datasets without further investigation is advisable. Furthermore, the augmentation of fit intervals to crack propagation rates smaller than $da/dN_{\mathrm{th}}$ may lead to non-conservative results and therefore it is not recommended.

If a valid threshold behavior is observed, the evaluation using the three-parameter polynomial Equation (5) provided constantly conservative, but not overly conservative, results that are neither sensitive to the data density nor to scatter. Therefore, we recommend this function over the linear fit over the first *n* points, which indeed performed well on regular shaped datasets. The fitting intervals shall follow the recommendations provided in the standards ($10^{-7}\;\mathrm{mm}/\mathrm{cycle}\leq da/dN\leq 10^{-6}\;\mathrm{mm}/\mathrm{cycle}$ for ASTM and $10^{-8}\;\mathrm{mm}/\mathrm{cycle}\leq da/dN\leq 10^{-7}\;\mathrm{mm}/\mathrm{cycle}$ for ISO). In case no definite threshold is reached, i.e., no crack arrest is observed (due for instance to anti-shielding effects, see Figure 11a), $\Delta K_{\mathrm{th}}$ cannot be determined. Therefore, we recommended providing the last da/dN- ΔK data pair for pure orientation, which shall not be intended as substitute for $\Delta K_{\mathrm{th}}$.

### 3.5. Application of the Fitting Methods to the IBESS Dataset

The proposed method has been validated further against the data from the IBESS project [23,24].Within the IBESS project, fatigue crack propagation tests have been performed on two different structural steels, S355NL and S960QL, using both standard *K*-decreasing procedures and CPLR tests at constant load ratios varying between R=−1 and R=0.7. The number of specimens tested at each stress ratio was limited; therefore, the present validation considered just those tests for which a meaningful data analysis could be performed. In particular, three tests at R=0 for the S355NL and three tests at R=0.5 for the S960QL have been considered. Furthermore, according to the recommendations on the fitting intervals given in this work, the data have been further narrowed. In case of the S355NL, all three datasets have been considered valid with respect to the ASTM fitting interval, whereas just two among them could be used for the determination of the fatigue crack propagation threshold according to ISO. For the S960QL, only two tests for ASTM and one for ISO have been included in the analysis. The results displayed in Table 12 and Table 13 confirm the conclusions drawn for the S690QL: the linear fit using the first *n* points and the polynomial with three parameters reduce the conservativeness in the evaluation of the fatigue crack propagation thresholds. The method proposed in the standards always provides the most conservative results. Furthermore, for the datasets with enough valid data in the fitting interval ($\Delta K_{\mathrm{th},\mathrm{ASTM}}$), the polynomial with three parameters provided the smallest standard deviation.

## 4. Conclusions

The present paper compared several methods for the evaluation of the fatigue crack propagation thresholds. New fitting strategies have been introduced and calibrated on a large dataset of crack growth data for the S690QL. The goodness of the fitting methods has been validated further against a dataset for S355NL and S960QL.

The following conclusions can be drawn:

- The ASTM E647 and ISO 12108 standards suggest to fit logΔK over logda/dN data using a linear fit, but leave plenty of room for interpretation with respect to the choice of the points in the fitting interval;
- When using all data points within the suggested fitting intervals, the most conservative values of $\Delta K_{\mathrm{th}}$ are obtained. However, the fit is not very subjected to scattered data;
- To use only the first *n* data points starting from the threshold crack propagation rate in order to ensure the best linear fit reduces the conservativeness at the cost of a more pronounced susceptibility to scatter and lower density of the data;
- The proposed fitting polynomials provided an improvement with respect to the goodness of the fit and susceptibility to scatter;
- An extrapolation of data was possible within given bounds for the structural steel S690QL, tested in lab air at room temperature at R≈0.8. Further tests comprising changes in materials, temperatures and the test environment should be conducted to assess the validity ranges;
- Tests subjected to crack closure phenomenon cannot be assessed in a fully automatic manner and require a manual dataset evaluation.

## Figures and Tables

**Table 1:** Chemical composition (in weight percent) obtained by means of spark optical emission spectrometry.

C	Si	Mn	P	S	Cr	Mo	Ni	Al	Cu	Nb	Fe
0.16	0.23	1.15	0.01	<0.01	0.41	0.18	0.04	0.08	0.02	0.04	97.65

**Table 2:** Mechanical properties.

$\sigma_{y}$ in MPa	$\sigma_{u}$ in MPa	*E* in GPa	*A* in %	${\mathrm{KV}}_{2}$ in J (Orientation: T–L [20])
810	825	207	16	126

**Table 3:** Comparison of effective threshold values for S690QL obtained with various fit methods. Tests conducted using the *K*-decreasing procedure at R=0.8 in lab air.

Method	$\Delta K_{\mathrm{th},\mathrm{ASTM}}$ in $\mathrm{MPa}\cdot m^{1/2}$	$\Delta K_{\mathrm{th},\mathrm{ISO}}$ in $\mathrm{MPa}\cdot m^{1/2}$
Linear, all data points (Equation (1))	2.71±0.06	2.27±0.04
Linear, first *n* data points (Equation (1))	2.76±0.05	2.29±0.04
Polynomial, neg. exp. (Equation (5))	2.78±0.04	2.34±0.04
Polynomial, neg. exp. $P_{3}=4$ (Equation (6))	2.77±0.05	2.32±0.04
Polynomial, neg. exp. $P_{3}=5$ (Equation (7))	2.78±0.05	2.32±0.03

**Table 4:** Comparison of effective threshold values for S690QL obtained with various fit methods. Tests conducted using the constant $K_{\max}$ procedure ($R_{\max}\approx 0.8$) in lab air.

Method	$\Delta K_{\mathrm{th},\mathrm{ASTM}}$ in $\mathrm{MPa}\cdot m^{1/2}$	$\Delta K_{\mathrm{th},\mathrm{ISO}}$ in $\mathrm{MPa}\cdot m^{1/2}$
Linear, all data points (Equation (1))	2.67±0.03	2.22±0.01
Linear, first *n* data points (Equation (1))	2.77±0.03	2.24±0.01
Polynomial, neg. exp. (Equation (5))	2.79±0.03	2.29±0.03
Polynomial, neg. exp. $P_{3}=4$ (Equation (6))	2.77±0.03	2.27±0.02
Polynomial, neg. exp. $P_{3}=5$ (Equation (7))	2.79±0.03	2.27±0.02

**Table 5:** Comparison of threshold values for S690QL obtained with various fit methods. Tests conducted using Compression Precracking Load Reduction at R=0.8 in lab air.

Method	$\Delta K_{\mathrm{th},\mathrm{ASTM}}$ in $\mathrm{MPa}\cdot m^{1/2}$	$\Delta K_{\mathrm{th},\mathrm{ISO}}$ in $\mathrm{MPa}\cdot m^{1/2}$
Linear, all data points (Equation (1))	2.67±0.02	2.22
Linear, first *n* data points (Equation (1))	2.74±0.03	2.23
Polynomial, neg. exp. (Equation (5))	2.77±0.04	2.29
Polynomial, neg. exp. $P_{3}=4$ (Equation (6))	2.73±0.02	2.26
Polynomial, neg. exp. $P_{3}=5$ (Equation (7))	2.74±0.03	2.27

**Table 6:** Comparison of threshold values for S690QL obtained with various fit methods. Tests conducted using ΔF-constant at R=0.8 in lab air.

Method	$\Delta K_{\mathrm{th},\mathrm{ASTM}}$ in $\mathrm{MPa}\cdot m^{1/2}$	$\Delta K_{\mathrm{th},\mathrm{ISO}}$ in $\mathrm{MPa}\cdot m^{1/2}$
Linear, all data points (Equation (1))	2.58±0.03	2.18±0.06
Linear, first *n* data points (Equation (1))	2.64±0.03	2.21±0.06
Polynomial, neg. exp. (Equation (5))	2.69±0.02	2.31±0.02
Polynomial, neg. exp. $P_{3}=4$ (Equation (6))	2.67±0.02	2.25±0.03
Polynomial, neg. exp. $P_{3}=5$ (Equation (7))	2.69±0.02	2.25±0.02

**Table 7:** Comparison of extrapolation errors obtained with various fit methods. The data refer to *K*-decreasing tests at R=0.8 and $K_{\max}$ tests conducted on S690QL in lab air. The evaluation comprised ten tests for ASTM threshold and thirteen specimens for ISO threshold. All values are given in $\mathrm{MPa}\cdot m^{1/2}$.

	$\Delta K_{\mathrm{th},\mathrm{ASTM}}-\Delta K_{\mathrm{th},\mathrm{cens},\mathrm{ASTM}}$		$\Delta K_{\mathrm{th},\mathrm{ISO}}-\Delta K_{\mathrm{th},\mathrm{cens},\mathrm{ISO}}$	
Method	$\mathrm{mean}\left(\;\cdot \;\right)$	$\min\left(\;\cdot \;\right)$	$\mathrm{mean}\left(\;\cdot \;\right)$	$\min\left(\;\cdot \;\right)$
Linear, all data points (Equation (1))	0.20	−0.17	0.07	−0.02
Linear, first *n* data points (Equation (1))	0.23	−0.18	0.07	−0.02
Polynomial, neg. exp. (Equation (5))	0.04	−0.02	0.03	−0.05
Polynomial, neg. exp. $P_{3}=4$ (Equation (6))	0.03	−0.01	0.03	−0.00
Polynomial, neg. exp. $P_{3}=5$ (Equation (7))	0.01	−0.02	0.02	−0.02

**Table 8:** Comparison of threshold values obtained at R≈0.8 with various fit methods using all 29 specimen data sets for $\Delta K_{\mathrm{th},\mathrm{ASTM}}$ and $\Delta K_{\mathrm{th},\mathrm{ISO}}$; results for S690QL in lab air.

Method	$\Delta K_{\mathrm{th},\mathrm{ASTM}}$ in $\mathrm{MPa}\cdot m^{1/2}$	$\Delta K_{\mathrm{th},\mathrm{ISO}}$ in $\mathrm{MPa}\cdot m^{1/2}$
Linear, all data points (Equation (1))	2.67±0.06	2.22±0.04
Linear, first *n* data points (Equation (1))	2.73±0.08	2.24±0.05
Polynomial, neg. exp. $P_{3}=4$ (Equation (6))	2.76±0.05	2.28±0.04

**Table 9:** Comparison of threshold values determined from artificial scattered test data, obtained at R≈0.8 with various fit methods using all 29 specimen data sets for $\Delta K_{\mathrm{th},\mathrm{ASTM}}$ and $\Delta K_{\mathrm{th},\mathrm{ISO}}$; results for S690QL in lab air.

Method	$\Delta K_{\mathrm{th},\mathrm{ASTM}}$ in $\mathrm{MPa}\cdot m^{1/2}$	$\Delta K_{\mathrm{th},\mathrm{ISO}}$ in $\mathrm{MPa}\cdot m^{1/2}$
Linear, all data points (Equation (1))	2.65±0.06	2.16±0.08
Linear, first *n* data points (Equation (1))	2.68±0.07	2.23±0.05
Polynomial, neg. exp. $P_{3}=4$ (Equation (6))	2.76±0.07	2.27±0.04

**Table 10:** Comparison of threshold values obtained with various fit methods in case of *K*-decreasing tests conducted on S690QL at R=−1 in lab air.

Method	$\Delta K_{\mathrm{th},\mathrm{ASTM}}$ in $\mathrm{MPa}\cdot m^{1/2}$	$\Delta K_{\mathrm{th},\mathrm{ISO}}$ in $\mathrm{MPa}\cdot m^{1/2}$
Linear, all data points (Equation (1))	13.43±0.91	12.78
Linear, first *n* data points (Equation (1))	13.67±0.80	12.89
Polynomial, neg. exp. (Equation (5))	13.69±0.82	12.92
Polynomial, neg. exp. $P_{3}=4$ (Equation (6))	13.56±0.85	12.90
Polynomial, neg. exp. $P_{3}=5$ (Equation (7))	13.57±0.85	12.90

**Table 11:** Comparison of threshold values obtained with various fit methods in case of compression precracking load reduction tests carried out at R=−1 in lab air.

Method	$\Delta K_{\mathrm{th},\mathrm{ASTM}}$ in $\mathrm{MPa}\cdot m^{1/2}$
Linear, all data points (Equation (1))	9.88±0.99
Linear, first *n* data points (Equation (1))	10.52±0.89
Polynomial, neg. exp. (Equation (5))	10.54±0.91
Polynomial, neg. exp. $P_{3}=4$ (Equation (6))	10.20±0.94
Polynomial, neg. exp. $P_{3}=5$ (Equation (7))	10.22±0.93

**Table 12:** Comparison of the threshold values for the S355NL tested at R=0 obtained with various fitting methods—a total of three sets are eligible for $\Delta K_{\mathrm{th},\mathrm{ASTM}}$ evaluation and two for $\Delta K_{\mathrm{th},\mathrm{ISO}}$.

Method	$\Delta K_{\mathrm{th},\mathrm{ASTM}}$ in $\mathrm{MPa}\cdot m^{1/2}$	$\Delta K_{\mathrm{th},\mathrm{ISO}}$ in $\mathrm{MPa}\cdot m^{1/2}$
Linear, all data points (Equation (1))	5.96±0.40	5.75
Linear, first *n* data points (Equation (1))	6.02±0.38	5.86
Polynomial, neg. exp. (Equation (5))	6.08±0.33	5.88

**Table 13:** Comparison of the threshold values for the S960QL tested at R=0.5 obtained with various fitting methods—a total of two sets eligible for $\Delta K_{\mathrm{th},\mathrm{ASTM}}$ evaluation and one for $\Delta K_{\mathrm{th},\mathrm{ISO}}$.

Method	$\Delta K_{\mathrm{th},\mathrm{ASTM}}$ in $\mathrm{MPa}\cdot m^{1/2}$	$\Delta K_{\mathrm{th},\mathrm{ISO}}$ in $\mathrm{MPa}\cdot m^{1/2}$
Linear, all data points (Equation (1))	3.30	3.04
Linear, first *n* data points (Equation (1))	3.31	3.10
Polynomial, neg. exp. (Equation (5))	3.31	3.08

## Data Availability

Not applicable.

## Symbols and Abbreviations

The following symbols and abbreviations are used in this manuscript:

ΔF	applied force range
ΔK	applied stress intensity factor range
$\Delta K_{\mathrm{LR}}$	*K*-decreasing FCG test procedure at constant load ratio
$\Delta K_{\mathrm{th}}$	fatigue crack propagation threshold
$\Delta K_{\mathrm{th},\mathrm{ASTM}}$	$\Delta K_{\mathrm{th}}$ referring to the ASTM operational definition
$\Delta K_{\mathrm{th},\mathrm{ISO}}$	$\Delta K_{\mathrm{th}}$ referring to the ISO operational definition
$\Delta K_{\mathrm{th},\mathrm{cens}}$	$\Delta K_{\mathrm{th}}$ obtained for the censoring of the data set
μ	mean value of the distribution
$\sigma_{u}$	ultimate tensile strength
$\sigma_{y}$	upper yield strength
*a*	crack size
*A*	elongation at break
da/dN	fatigue crack propagation rate
$da/dN_{\mathrm{th}}$	da/dN referring to an operational threshold definition
$da/dN_{\mathrm{th},\mathrm{ASTM}}$	ASTM operational threshold definition of $da/dN_{\mathrm{th}}$
$da/dN_{\mathrm{th},\mathrm{ISO}}$	ISO operational threshold definition of $da/dN_{\mathrm{th}}$
*e*	Weibull threshold parameter
*E*	Young’s modulus
*I*	degree of the polynomial
*k*	Weibull shape parameter
$K_{b}$	Weibull instability parameter
$K_{\max}$	maximum stress intensity factor in a loading cycle
$KV_{2}$	impact energy
*n*	number of data points
*N*	number of loading cycles
$P_{i}$	fitting parameters, i∈N
*R*	stress ratio
$R_{\max}$	maximum stress ratio within $K_{\max}$-FCG test
*v*	Weibull characteristic value
ASTM	American Society for Testing and Materials
BAM	Bundesanstalt für Materialforschung und -prüfung
CPLR	compression precracking load reduction
FCG	fatigue crack growth
ISO	International Organization for Standardization
MPA-IfW	Materialprüfungsanstalt Darmstadt, Institut für Werkstoffkunde
ODR	orthogonal distance regression
SD	standard deviation
SENB	single edge notch bending
